# Compressive Stimulation Enhances Ovarian Cancer Proliferation, Invasion, Chemoresistance, and Mechanotransduction via CDC42 in a 3D Bioreactor

**DOI:** 10.3390/cancers12061521

**Published:** 2020-06-10

**Authors:** Caymen M. Novak, Eric N. Horst, Emily Lin, Geeta Mehta

**Affiliations:** 1 Biomedical Engineering, University of Michigan, Ann Arbor, MI 48109, USA; cmnovak@umich.edu (C.M.N.); horster@umich.edu (E.N.H.); emilylin@umich.edu (E.L.); 2 Materials Science and Engineering, University of Michigan, Ann Arbor, MI 48109, USA; 3 Macromolecular Science and Engineering, University of Michigan, Ann Arbor, MI 48109, USA; 4 Rogel Cancer Center, University of Michigan, Ann Arbor, MI 48109, USA; 5 Precision Health, University of Michigan, Ann Arbor, MI 48109, USA

**Keywords:** mechanotransduction, ovarian cancer, bioreactor, compression, three dimensional, hydrostatic pressure, ovarian tumors, hydrogels, compressive stress, high grade serous

## Abstract

This report investigates the role of compressive stress on ovarian cancer in a 3D custom built bioreactor. Cells within the ovarian tumor microenvironment experience a range of compressive stimuli that contribute to mechanotransduction. As the ovarian tumor expands, cells are exposed to chronic load from hydrostatic pressure, displacement of surrounding cells, and growth induced stress. External dynamic stimuli have been correlated with an increase in metastasis, cancer stem cell marker expression, chemoresistance, and proliferation in a variety of cancers. However, how these compressive stimuli contribute to ovarian cancer progression is not fully understood. In this report, high grade serous ovarian cancer cell lines were encapsulated within an ECM mimicking hydrogel comprising of agarose and collagen type I, and stimulated with confined cyclic or static compressive stresses for 24 and 72 h. Compression stimulation resulted in a significant increase in proliferation, invasive morphology, and chemoresistance. Additionally, CDC42 was upregulated in compression stimulated conditions, and was necessary to drive increased proliferation and chemoresistance. Inhibition of CDC42 lead to significant decrease in proliferation, survival, and increased chemosensitivity. In summary, the dynamic in vitro 3D platform developed in this report, is ideal for understanding the influence of compressive stimuli, and can be widely applicable to any epithelial cancers. This work reinforces the critical need to consider compressive stimulation in basic cancer biology and therapeutic developments.

## 1. Introduction

Ovarian cancer is subject to a variety of pathophysiological mechanical forces during initiation, progression, and metastasis [1]. Primary ovarian tumors reach average diameters of 12 cm, displacing surrounding extracellular matrix and native cells [2,3]. This uncontrolled growth produces a circumferential compressive force on the tumor [4]. Additionally, ovarian cancer patients often present with ascites, or the retainment of fluid within the peritoneal cavity. This fluid build-up submerges the ovaries in an aberrant mechanical microenvironment that further compresses the primary tumor site through hydrostatic pressure. As patients go about their everyday routine, the movement and placement of the ascites alters hydrostatic pressure in a time dependent manner [5,6]. This resulting compression stimulates the cells and impacts downstream signaling, known as mechanotransduction.

Mechanotransduction has been shown to influence the progression of cells and cell fate in a variety of biological systems. When studied independently, compression [7], tension [8], matrix stiffness [9], and shear stress [10] have all been attributed to the altered proliferation and survival of cancer cells. Responses to these physiological stimuli are widespread and ingrained into cell fate and phenotypic responses [11]. In solid tumors, growth-induced stress is predicted to reach 18.9 kPa—albeit these measurements omit the impact of the surrounding extracellular matrix (ECM), and are likely higher in situ [12]. Gene expression pathways shown to be influenced by the compressive environment include those linked to cell death, proliferation, cell attachment, and epithelial to mesenchymal transition (EMT) [7,13,14]. Homeostatic cells typically halt proliferation when they sense neighboring structures or are restrained by compressive duress [15]. However, the specific cellular response varies depending on force magnitude, duration, and method of application. In the diseased state, cancer cells under compressive stimulation have been shown to alter their proliferation potential, though contradictory findings may indicate context dependency [16,17,18]. Compression has been shown to be an ineffective regulator of proliferation in cancer, whereby dysregulation of the CHK2-p53 pathway blocks compression-mediated S phase arrest [17]. The application of compressive force induces cell death in breast cancer through a mixed apoptotic and necrotic cell program [19]. Despite conflicting evidence regarding proliferation, the tendency of compressive forces to drive infiltration and thus metastasis has been well documented in several cancer cell types. In breast cancer, compression leads to the development of leader cells and invasive phenotypes [13]. In pancreatic and brain cancers, compression induces migration, arbitrated by GDF15 through the Akt [20] and the MEK1/Erk1 pathways respectively [14]. Tumor microenvironment compression-induced solid stress has been linked to alternative activation of surrounding stromal cells including fibroblasts which further promote cancer cell migration [21]. Taken together, solid tumor stress seems to differentially affect cancer types, depending on tumor heterogeneity and oncogenic mutation, understanding the response of compressive stimulation in cancer is critical for advancing patient treatment.

Cells stimulated with compressive stress rely on mechanotransduction via modulation of cytoskeletal deformation [22], integrin binding complexes [23], ion channels [24], and lipid rafts [25]. Rho family GTPase cell division cycle 42 (CDC42) links mechanical sensitive focal adhesions to protrusive filopodia and acts as a molecular switch for a variety of cellular processes [26,27]. CDC42 affects over 20 downstream pathways, and is influenced by over 30 promoters, inhibitors, and upstream regulators [28]. Its overexpression is prevalent in a variety of cancers where it has been shown to influence proliferation, motility, polarity, growth, filopodia formation [29], vesical trafficking, transcription, and cytokinesis [28]. Its role in mechanotransduction has been tied to matrix stiffness [30] as well as tension induced YAP pathway activation [31], a well-known mechanoresponsive cell signaling cascade [32]. CDC42 signaling in ovarian cancer has been associated with disease progression [33] and is frequently overexpressed in primary ovarian tumors [34], though its association with compressive stimulation is still unknown.

Within ovarian cancer, the influences of mechanotransduction are not thoroughly understood, and while a majority of studies focus on fluid shear stress, compressive stress remains relatively unexplored [35,36]. One study mimicking the compressive forces present within patient ascites, found that compressive stress upregulates EMT markers in cellular aggregates of ovarian cancer [5]. These results highlight that compressive stress in ovarian cancer remains an area of research in urgent need for fundamental study. Therefore, in this report, we utilized a tunable 3D compression bioreactor to systematically test high grade serous ovarian cancer cells in 24 and 72 h loading regimes using a custom-built in-house bioreactor. The cells were encapsulated within an interpenetrating 3D agarose-collagen type I hydrogel. Morphological changes, gene expression profiles, alongside proliferation and cell death were monitored in response to compressive stimuli. We identify mechanosensitive regulators necessary to elicit these responses in high grade serous ovarian cancer cell lines. This study underscores the relevance of mechanical stimulation, in ovarian cancer, and their role in disease progression and treatment.

## 2. Results

### 2.1. COMSOL Compression Bioreactor Model Shows Pressure Distribution Within Cell Laden Interpenetrating Hydrogel

**Figure 1:**
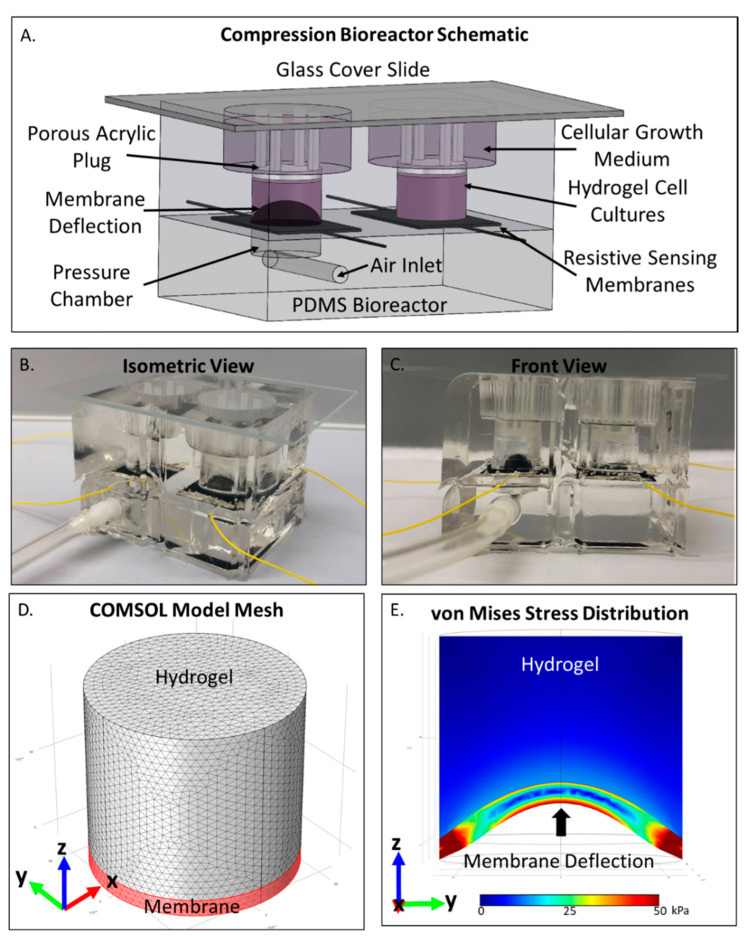
Ovarian cancer compression bioreactor schematic and computational model. (**A**) SolidWorks rendering of the compression bioreactor. Air is pumped into the underlying pressure chamber which deflects into the cancer cell laden interpenetrating hydrogel. The 3D cellular hydrogel component is held in place via a porous acrylic plug which also allows cell culture medium access from the top of this chamber; (**B**) Isometric view of a representative compression bioreactor; (**C**) Front view of a representative compression bioreactor with air pressure applied to demonstrate deflection of resistivity sensing membrane (black dome); (**D**) Mesh construction on COMSOL model of the hydrogel (1 cm height by 6 mm radius) and deflectable membrane (1 mm height by 6mm radius); (**E**) Sample output of COMSOL analysis showing center slice through z-y plane. Deformation of the membrane and hydrogel are shown with an applied pressure of 20 kPa. Average compressive stress within the hydrogel is 5.2 kPa.

With an aim of predicting the compressive forces experienced by high grade serous ovarian cancer cells within the compression bioreactor (Figure 1A,B), a COMSOL model of the cell-laden interpenetrating hydrogel and force applying deflection membrane was constructed. A sample mesh of the model is provided in Figure 1D and the corresponding mesh analysis can be found in Figure S1. Von Mises stress distribution throughout the hydrogel was found to apply an average static compressive force of 5.2 kPa to encapsulated cells and vary from 3.9–6.5 kPa under cyclic loading regimens (Figure 1E). Although greater compressive forces were observed near the contacting plane of the hydrogel-membrane, cellular responses were averaged throughout the entirety of the hydrogel. Therefore, the output compressive stimulation was reported as the average Von Mises stress throughout the 3D cellular microenvironment.

### 2.2. Compressive Stimulation Induces Invasive Morphology in High Grade Serous Ovarian Cancer Cells

**Figure 2:**
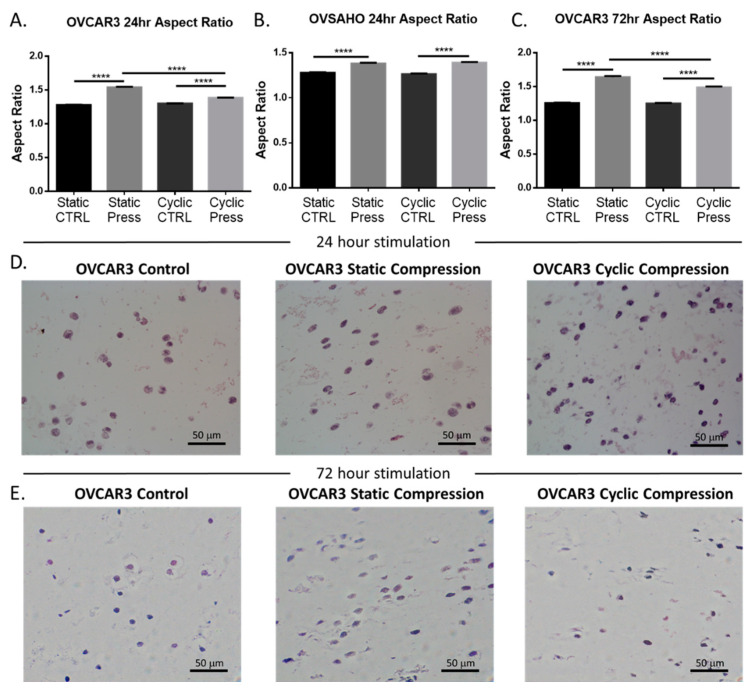
Cyclic and static compressive stresses cause significant morphological changes in high grade serous ovarian cancer cells indicating increased invasive potential. (**A**) 24 h compressive stimulation of OVCAR3 cells under static or cyclic loading; (**B**) 24 h compressive stimulation of OVSAHO cells under static or cyclic loading; (**C**) 72 h compressive stimulation of OVCAR3 cells under static or cyclic loading; (**D**) Representative images of ovarian cancer cellular morphology under control, static, or cyclic loading conditions at 24; (**E**) and 72 h. (Significance calculated via one-way ANOVA; *n* ≥ 3 experimental replicates, **** *p* < 0.0001, *** *p* < 0.001, ** *p* < 0.01, * *p* < 0.1).

The shape of a cell is a known indicator of cellular fate and migratory potential and intention [37]. Therefore, morphological analysis was performed to evaluate the influence of compressive stimulus via hematoxylin and eosin (H and E) staining. Cellular morphology was significantly altered in response to compressive stimulus. Cells displayed an increase in aspect ratio across cell type and duration of compression application (Figure 2). A significant increase in aspect ratio was also seen in statically compressed vs. cyclically compressed OVCAR3 cells for both the 24 h and 72 h timepoints. Additional quantification of the cellular area, perimeter, circularity, and roundness can be found in Figure S2. The elongation of the ovarian cancer cells, as observed in these data, is indicative of an invasive phenotype and likelihood of their metastasis [38].

### 2.3. Compression Enhances High Grade Serous Ovarian Cancer Cell Proliferation and Reduces Cell Death

**Figure 3:**
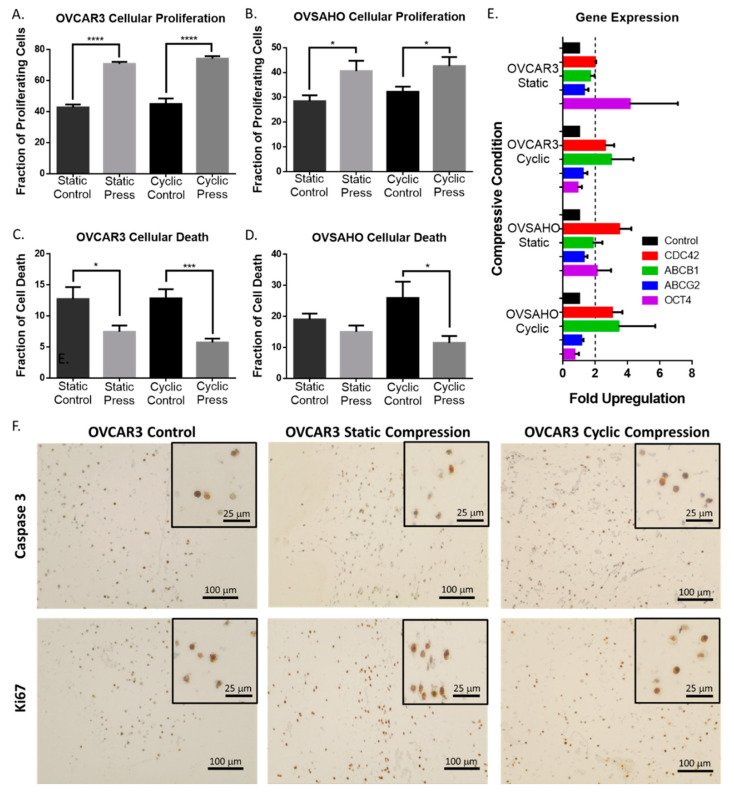
Compressive stimulation of ovarian cancer cells causes significant changes in proliferation, cell death, and gene regulation. Cellular proliferation of (**A**) OVCAR3 and (**B**) OVSAHO cells under static and cyclic compressive stress stimulation for 24 h (IHC ki67 expression). Cell death of (**C**) OVCAR3 and (**D**) OVSAHO cells under static and cyclic compressive stress stimulation for 24 h (IHC cleaved caspase 3 expression). (Significance calculated via *t*-test; *n* ≥ 3 experimental replicates, **** *p* < 0.0001, *** *p* < 0.001, ** *p* < 0.01, * *p* < 0.1). (**E**) Gene expression changes via RT-qPCR for ovarian cancer cells stimulated for 24 h under static or cyclic compressive conditions. A two-fold upregulation is indicated by the dotted line. (**F**) Representative IHC images of OVCAR3 expression of cleaved caspase 3 and ki67 under compressive stress stimulation for 24 h.

Changes in cellular morphology are an established modulator of cellular proliferation and survival [37] and a critical component to cancer progression [38]. Thus, the proliferation and cell death phenotype in response to cyclic and static compressive stimulation was evaluated in ovarian cancer cells. Cells subjected to compressive stimulation displayed a significant increase in proliferation marker ki67, as well as a reduction in cell death marker cleaved caspase-3 (Figure 3A–D). This trend was sustained for all forms of compressive stimulation in both high grade serous cell lines (Figure 3), although these trends were not significantly maintained for the 72 h time point (Figure S3). 

### 2.4. Compressive Stimulation of High Grade Serous Ovarian Cancer Cells induces Overexpression of CDC42

To understand the mechanism by which compressive stress induced mechanotransduction may be regulating alterations in proliferation, cell death, and morphology, a RT-qPCR analysis was performed on a wide variety of genes known to be involved in mechanotransduction, metastasis, cancer stem cells, EMT, and ovarian cancer. A significant upregulation of CDC42 was found in both cell types for both static and cyclic compressive stimulus (Figure 3E). Additionally, upregulation of known chemotherapeutic efflux pumps, ABCB1 and ABCG2, was observed when compared to non-stimulated controls though this change was not significant in all conditions. Interestingly, significant upregulation of stem cell marker OCT4 was observed in both cell types but only under static compression conditioning, indicating a stimulus specific response to compression loading regimes (Figure 3E). 

### 2.5. Chemoresistance in High Grade Serous Ovarian Cancer Cells Is Observed Under Compressive Stimulation

**Figure 4:**
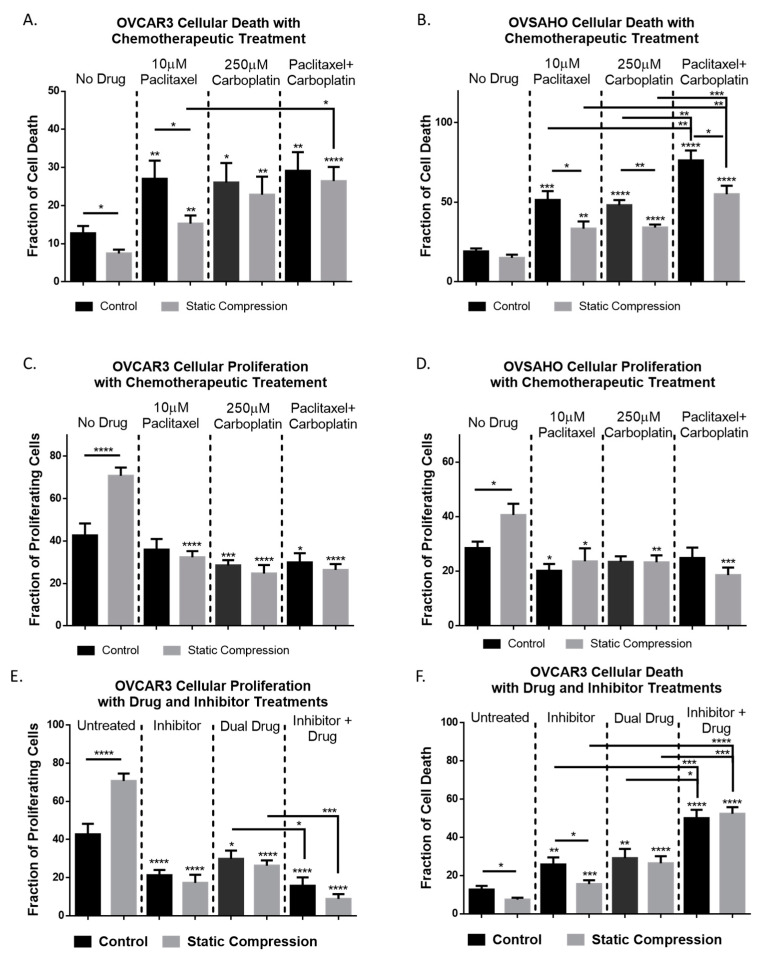
Compressive stimulation of ovarian cancer cells causes chemoresistance which can be mitigated through concurrent ML141 100 μM treatment over 24 h. Star indicators located directly above a column indicates significant change when compared to the non-drug treated control/compression condition respectively. (**A**) OVCAR3 cellular death rates in response to paclitaxel, carboplatin, and combination treatments. Significant reduction in cell death is observed under compressive paclitaxel treatment. (**B**) OVSAHO cellular death response to chemotherapeutic treatments. Significant reduction in cell death is observed in all compression conditions when compared to non-stimulated drug-treated controls. (**C**) OVCAR3 proliferation responses to chemotherapeutic treatments. Drug treatments effectively target enhanced proliferation caused by compressive stimulus. (**D**) OVSAHO proliferation responses to chemotherapeutic treatments show compression induced proliferation enhancement effectively mitigated by drug administration. (**E**) OVCAR3 proliferation response to CDC42 inhibitor treatment, dual drug chemotherapy, and combined treatment. (**F**) OVCAR3 cellular death rates in response to CDC42 inhibitor treatment, dual drug treatment, and combination therapy. Combination therapy more effectively targets proliferating cells and improves cell death count compared to either treatment alone. (Significance calculated via *t*-test; n ≥ 3 experimental replicates, **** *p* < 0.0001, *** *p* < 0.001, ** *p* < 0.01, * *p* < 0.1).

Given the upregulation of chemoresistance genes observed in the RT-qPCR array, investigation of the cellular response to clinically used chemotherapeutics paclitaxel and carboplatin was performed. A slight reduction in cell death was observed for OVCAR3 cells under compression when treated with independent or dual drug treatment though only paclitaxel showed a significant reduction in cell death of cells under compression (Figure 4A). OVSAHO cells showed a significant reduction in cell death in response to chemotherapy for all treatment regiments when under compression indicating chemoresistance (Figure 4B). Cellular proliferation for both cell lines was significantly reduced with chemotherapeutic treatment and no significant difference between compression loaded and unloaded samples was observed (Figure 4C, D). 

### 2.6. Inhibition of CDC42 in High Grade Serous Ovarian Cancer Cells Reduces Compression-Induced Proliferation, Cell Survival, and Chemoresistance

Given the upregulation of CDC42 observed under compressive stimulation, we hypothesized that the phenotypic changes observed in ovarian cancer cells under compression rely on CDC42 activity. CDC42 activation was first quantified using the Cytoskeleton G-lisa assay (Figure S4) which confirmed the increased level of activated GTP bound CDC42 under compressive stimulus. We then investigated the effective use of CDC42 specific nucleotide binding inhibitor ML141 which showed a 70% reduction in active GTP bound CDC42 with 100 µM treatment (Figure S4).

Since CDC42 is such a widespread effector and is a known influencer of cell cycle progression we investigated the impact of inhibition on proliferation and cell death under compressive stimulus. IHC staining displayed a significant reduction in cellular proliferation as well as an increase in cell death for both stimulated and control cells alike (Figure 4E,F). Cellular death was significantly lower in inhibited-compression samples compared to inhibited-control samples though both cellular death levels were significantly increased when compared to non-inhibited controls (Figure 4F). 

With combination therapy of dual drug treatment and ML141, a significant increase in cell death was observed compared to either treatment independently (Figure 4F). Additionally, the number of proliferating cells was significantly reduced with combination therapy compared to dual drug treatment alone (Figure 4E). This indicates the chemoresistant effect observed under compressive stress can be overcome and treatment effectiveness may be enhanced through combination therapy when compared to chemotherapeutics alone. 

### 2.7. High Grade Serous Ovarian Cancer Cellular Area Is Significantly Increased With Inhibited CDC42 and Combination Chemotherapy 

Given that CDC42 has been identified as a contributor to cellular morphology [39], we investigated the simultaneous effect of CDC42 inhibition and drug treatment on cell shape (Figure S5). The most significant changes were observed with simultaneous inhibitor and drug treatment, with cellular area significantly increasing for both control and compressed cells. However, under compressive stimulation, the aspect ratio of the cells was maintained significantly higher than controls for dual drug treatment, inhibitor treatment, and combination therapy (Figure S5) implying compression induced invasive morphology is not dictated by CDC42 activation. 

## 3. Discussion

Ovarian cancer cells experience a unique mechanical microenvironment subjected to both transient and static compressive forces, an aspect of this deadly gynecologic disease that has yet to be thoroughly explored. The mechanical forces that ovarian cancer cells experience play a major role in their fate. Therefore, understanding how mechanotransduction influences ovarian cancer progression will lead to the creation of future treatments that target the mechanical microenvironment, inhibit mechanotransduction, or disrupt key regulators of aberrant mechanical sensors. In order to examine the understudied compressive mechanical microenvironment in ovarian cancers, we have designed and constructed a tunable 3D compression bioreactor. Within this custom-built compressive bioreactor, high grade serous ovarian cancer cells were embedded in an interpenetrating network hydrogel and subjected to cyclic and static compressive stresses. The role of the compressive stimuli on ovarian cancer proliferation, chemotherapy response, and morphological changes were quantified.

Computational modeling found an average compressive force of 5.2 kPa was applied to the cell laden IPN hydrogels. This stimulus value is on the lower end of predicted compressive forces experienced by the primary tumor in situ [12]. Static compressive stress of 5.2 kPa and cyclic compressive stress of 5.2 ± 1.6 kPa was applied for 24 to 72 h, increasing the proliferative potential of the high grade serous ovarian cancer cells, while decreasing overall cell death. These results imply a feed forward mechanism where tumor growth enhances compressive forces, further stimulating tumor proliferation. Previous studies concerning other cancer cell types under compressive stimulation did not observe changes in proliferative potential [13], indicating a unique phenotypic change for ovarian cancer cells. The morphological alteration of the cells under compressive stimulus for static and cyclic compressive stresses over 24 and 72 h of stimulation demonstrated elongation of the cells, indicating invasive potential. Surprisingly, there was no observable difference in ovarian cancer cellular proliferation between the cyclic and static compressive stress stimulations. However, static compressive stimulation enhanced the aspect ratio of the OVCAR3 cells, when compared to cyclic loading. Although both static and cyclic compressive stimulation significantly increased cellular elongation compared to 3D grown non-stimulated controls. These results indicate that the static and cyclic compressive stimulation similarly impact high grade serous ovarian cancer phenotypic changes under these loading conditions. 

CDC42 was identified as one of the important mediators of compressive stimulation-led mechanotransduction in high grade serous ovarian cancers. Significant CDC42 upregulation was observed in both high grade serous ovarian cancer cell types and compressive stimulation conditions (static and cyclic) compared to non-stimulated 3D controls. CDC42 has previously been proven to regulate mechanotransduction responses, such as endothelial migration [40,41], osteoblast β-catenin signaling [42], integrin signaling [43], morphology and differentiation of mesenchymal stem cells [44], and stiffness-induced dormancy of cancer cells [30]. However, the role of CDC42 in ovarian cancer compressive stress induced mechanotransduction was not identified before this report. 

Drug treatment responses under compressive stimulation demonstrated chemoresistance for high grade serous ovarian cancer cells. The clinically utilized combination treatment of paclitaxel and carboplatin significantly increased cell death in ovarian cancer cells compared to non-drug treated controls as expected, however, was less effective under compressive conditions. Specific CDC42 inhibition, through ML141 treatment, also significantly reduced the ovarian cancer cell proliferation and increased cancer cell death for both control and compressive stimulations. When CDC42 inhibition was combined with dual drug treatment, the ovarian cancer cell proliferation was significantly decreased, and cell death was enhanced beyond chemotherapeutics or inhibitor use alone. These findings support the proposed use of CDC42 inhibitors or alternative pathway interventions in combination with chemotherapy for improved patient treatments, as suggested for several other cancer types [45], though no known clinical trials for this combination therapy currently exist. 

**Figure 5:**
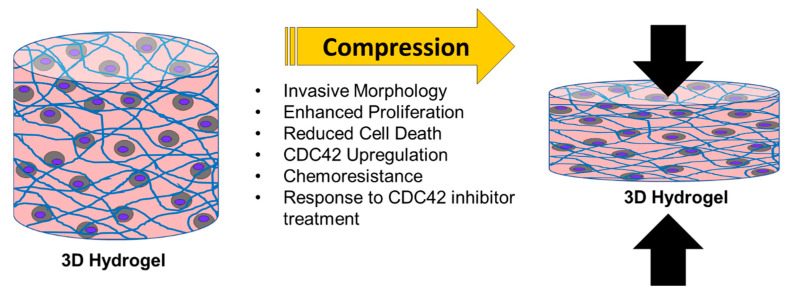
Summary of ovarian cancer response to compressive stimulus within a 3D agarose-collagen I hydrogel. Arrows indicate the applied compressive force (applied from the bottom) and its reactive force (represented by the top arrow). Ovarian cancer cells under compressive stimulus display an invasive morphology through their elongation and increase in aspect ratio. They also enhance proliferation and reduce overall cell death as shown in the immunohistochemical staining of ki67 and caspase-3 respectively. Compressive stimulus consistently upregulates CDC42 and chemoresistance to standard ovarian cancer drug treatments paclitaxel and carboplatin. Treatment with the CDC42 inhibitor ML141 facilitates chemotherapeutic response through enhanced cell death and reduced proliferation.

Taken together, these results demonstrate the physiological application of our custom-built 3D dynamic compression bioreactor for the investigation of mechanotransduction via compressive stimulation on ovarian cancer cells (Figure 5). Compressive stimulation significantly alters ovarian cancer cell phenotype and plays a major role in the progression of the disease. Dual treatment with CDC42 inhibitor along with combination chemotherapies, increases high grade serous ovarian cancer cell death, while reducing proliferation. Thus, future synergistic treatments that target tumor cells directly (via chemotherapies) and mechanotransduction pathways, will serve as a promising avenue for sustained clinical responses and reduced relapses. 

## 4. Materials and Methods 

### 4.1. Cell Culture

Cell culture reagents purchased from Thermo Fisher Scientific: RPMI growth medium (11875119), antibiotic/antimycotic (15240062), 0.25% trypsin-EDTA (25-200-056), rat tail collagen type I (344310001). Human ovarian cancer cell line OVCAR3 was purchased from American Type Culture Collection (ATCC, Manassas, VA, USA) and ovarian cancer cell line OVSAHO was kindly provided as a gift from the Buckanovich lab (Magee-Womens Research Institute, Pittsburgh, PA, USA). Low melt agarose was purchased from Boston Bioproducts Inc. (P73050G, Ashland, MA, USA). Chemotherapeutic drugs Paclitaxel (T7402) and carboplatin (C0171) was purchased from Sigma-Aldrich (St. Louis, MO, USA). Fetal bovine serum (FBS) was purchased from Gemini Bio-Products (100-106, West Sacramento, CA, USA).

Cells were plated on 15 cm polystyrene plates with 1640 RPMI cell culture medium containing 10% FBS and 1% anti-anti until 80% confluency was reached. Cell cultures were maintained until use in the compression bioreactor. Cells were collected from plates using 0.25% trypsin and pelleted before suspension at 10 million cells/mL in the interpenetrating hydrogel comprising of 3% agarose and 0.05% collagen type I, as previously described [10]. The cell laden hydrogels were plated within the control or experimental wells of the compression bioreactor and allowed to solidify. Finally, the entire device was placed within the cell culture incubator (5% CO_2_, 37 °C) for the duration of the experiment. 

### 4.2. Device Construction and Use

The following materials were used in the construction of the 3D compression bioreactor: PDMS (0007604765 Sylgard 184, Ellsworth Adhesives, Germantown, WI, USA), carbon nanotubes (CNT) (030104, Cheaptubes.com, Grafton, VT, USA), silver epoxy (8331-14G, Electronic Parts Specialist, Flint, MI, USA), 22 AWG single stranded wire (602-3051/1-100-01, Mouser Electronics, Mansfield, CA, USA), 1/8 inch male luer locks (5121K151, McMaster-Carr, Elmhurst, IL, USA), tubing (5195T62, McMaster-Carr). The compression bioreactor body was made from PDMS (1:8 curing agent to PDMS ratio) molded over metal constructs to create the negative cell + hydrogel wells, and air chambers as shown in Figure 1A–C in two parts. The upper half of the bioreactor includes the cell culture well and medium reservoir, while the lower portion contains the air pressure chamber and air inlet tube. The cell culture wells had six radial cuts, 3 mm deep, running from the base (contact with the CNT membrane) to the top (media reservoir) that aided cell culture medium diffusion to the entirety of the cell laden hydrogel. Deflecting membranes were generally modeled after the work done by MacQueen et al. [46], made from carbon nanotube and PDMS combined with a 1:10 curing agent weight ratio. The PDMS/curing agent/CNT mixture was then spin coated onto salinized glass slides to a thickness of 0.5 mm. The resulting membranes had a Young’s modulus of 248 kPa as determined through tensile testing on the TA XT Texture Analyzer (Figure S1)**.** The spin coated slides were cured at 40 °C overnight before use. Membranes were then checked for conductivity, removed from the glass slide, placed over the air pressure chamber and sealed to the PDMS mold of the lower air chamber using 1:8 PDMS and this was once again cured overnight at 40 °C. Silver epoxy was then used to connect wire to either end of the conductive membrane and allowed to cure. The upper PDMS construct was then fitted atop the constructed lower portion using 1:8 PDMS and allowed to cure. The double male ended tubing connector was fitted into the air inlet chamber and the entire construct was autoclaved for sterilization before use. To minimize air leakage through the PDMS components, the underlying pressure chamber was filled with water to deflect the CNT membrane during compression application. 

### 4.3. Electrical Hardware and Software Programming

LabVIEW programming was utilized to run and maintain desired compression functions. Compression application was monitored simultaneously through resistivity measurements, digital pressure sensor, and mechanical pressure sensor. The schematic of the entire compression bioreactor system is depicted in Figure S6.

The bioreactor was connected via tubing to the syringe pump actuator system which controlled the supplied pressure via linear actuator displacement of a syringe. The air pressure within the closed system was measured via a digital pressure gauge (Omega, Norwalk CT, USA) which was used within the LabVIEW program to monitor force application. 

A DAQ board (NI USB 6008) was used to interface with the pressure driving linear actuator (L12-I, Firgelli, Ferndale, WA, USA), pressure gauge, and LabVIEW software. Each compression bioreactor was conditioned for three thirty-minute regimens of cyclic pressure loading while monitoring the resistivity change in the membrane in order to get a run-in profile for each bioreactor. Sample resistivity response curves to static and cyclic pressure functions are provided in Figure S7. Resistance of the membrane was correlated to membrane deflection and applied pressure to monitor compression throughout the duration of the experiment. An amount of 20 kPa of maintained pressure was applied to static compression experiments and 20 ± 5 kPa of air pressure was cycled sinusoidally at a frequency of 0.05 Hz for cyclic compression experiments. 

### 4.4. COMSOL Computational Analysis

Hydrogel characteristics previously determined through SEM, porosimetery, and rheometry [10] were used as inputs for the COMSOL Multiphysics 5.3 computational analysis of applied compressive stress and are provided in Figure S1. Solid stress physics was used to describe the application force and resultant stresses. Linear elastic material was used to describe both the hydrogel and membrane characteristics and an underlying boundary load was used to define the pressure application on the membrane (Figure S1). 

### 4.5. Morphological Cell Analysis and Immunohistochemistry 

Cell-laden hydrogels were removed from the bioreactor and histologically processed for staining. Briefly, whole cell-gel constructs were removed from the compression bioreactor from either the control or compression wells. Next whole gels were cut in half along the z-y plane (Figure 1E). Next these pieces were fixed in formalin and paraffin embedded before sectioning into 5 μm slices. Hematoxylin and eosin staining was performed for morphological analysis, and slides were imaged using a Nikon E800 light microscope. Stained sections were partitioned into quadrants and a minimum of 3 representative images in each quadrant were taken at 40× magnification for analysis. Approximately 600 cells per condition were measured. Cellular morphology was quantified using a custom MATLAB R2019a program that detected and encompassed the perimeter of the cell and calculating cellular area, perimeter, circularity, roundness, and aspect ratio (Figure 2 and Figure S2). These results were then used for comparison and statistical analysis of each experimental condition.

Histological sections of experimental hydrogels were stained for cell death and proliferation quantification using a Casp-3 (PI700182, Fisher Scientific, Waltham, MA, USA) and Ki67 (PA5-16785, Fisher Scientific) antibody respectively according to VECTASTAIN Elite ABC-HRP Kit (PK-6101, Vector Laboratories, Burlingame, CA, USA) IHC staining procedure. Three images per section were taken and quantified using ImageJ cell counter plugin 2.2.2 [47]. Approximately 600 cells were analyzed per condition. 

### 4.6. Gene Expression Analysis

Cell laden hydrogels were removed from the bioreactor and processed using the Qiagen RNeasy Mini kit for digestion and RNA purification. An additional RPE was step was utilized to achieve necessary RNA purification. Gene expression changes were investigated through RT-qPCR for a variety of genes involved in metastasis, EMT, mechanotransduction, chemoresistance, and cancer stem cell markers. Primer pairs utilized for RT-qPCR are tabulated in Table S1. RT-qPCR was performed using 96 well plates and Power SYBR Green PCR master mix (ILT4367659) on a 7900HT system through the DNA sequencing core at the University of Michigan. Significance was determined through the 2^-ΔΔCT^ method comparing the compression stimulated cells to their corresponding controls [48]. Three biological replicates per condition were analyzed with three technical replicates per plate. Significance was defined as a two-fold change or greater from the controls. 

### 4.7. G-Lisa Assay (CDC42 Activation and Inhibition)

To assess CDC42 activation, a G-Lisa assay from Cytoskeleton Inc. (Cat. # BK127) was used. Sample preparation procedures were followed as directed in kit instructions with few modifications. The included: gels were pulverized while incubating in the lysis buffer, protein concentrations of 2.5 mg/mL were used, and incubation time in the 37 °C incubator was extended to 20 min. Inhibition of CDC42 was achieved through the specific CDC42 inhibitor ML141 (217708-25MG, Sigma-Aldrich, St. Louis, MO, USA) [49]. Reduction in CDC42 activation was monitored via the G-Lisa colorimetric assay and results are shown in Figure S4.

### 4.8. Chemotherapeutic Treatment

Drug treatment was performed using paclitaxel 10 μM and carboplatin 250 μM independently and in combination. Treatment concentrations were drawn from previously published studies on OVCAR3 cell death in response to carboplatin treatment [50] and paclitaxel treatment of epithelial ovarian cancer cell lines [51]. Paclitaxel was diluted in DMSO and carboplatin was suspended in water before addition to cell culture medium within the compression bioreactor. All chemotherapeutic drug experiments were performed on either control or statically loaded hydrogels. 

### 4.9. Statistical Analysis

Three or more biological replicates were performed for each experiment with three technical replicates in each run. Statistical analysis and graphical plots were constructed in GraphPad Prism 8 (GraphPad, San Diego, CA, USA) software using either one-way ANOVA followed by Kruskal–Wallis nonparametric test or *t*-test. All data are displayed as mean ± SEM. Significance in gene regulation was defined as greater than a twofold increase (2) or a twofold decrease (0.5) in expression.

## 5. Conclusions

In conclusion, we have developed a tunable 3D compression bioreactor for investigating ovarian cancer mechanotransduction. The bioreactor is capable of programmable compression functions, as well as static loading which is continuously monitored via LabVIEW programming. This self-maintaining system was shown to be useful for extended cultures up to 72 h and is widely applicable for a variety of cell types. The study of compressive stimulus on ovarian cancer outcomes has been largely neglected with this being the second reported disease specific compression study. Here we demonstrated that ovarian cancer cells under physiological compressive stimulus have enhanced invasive potential, increased proliferative capacity, reduced apoptosis, and increased chemoresistance. Additionally, ovarian cancer cells upregulate CDC42 significantly and consistently across high grade serous cell types and compressive stimulation type (static or cyclic). Inhibition of CDC42 resulted in the mitigation of enhanced proliferation, and resistance to chemotherapy, although invasive morphology was maintained. Further investigation into the mechanism by which high grade serous ovarian cancer cells experience compression and control downstream effectors of CDC42 will be an important step for the development of targeted therapies that prevent this apparent feed forward mechanism of stress induced proliferation and drug resistance. Overall, this work demonstrates the importance of physiological compressive stimuli on ovarian cancer mechanotransduction and its impact on treatment responses with combinatory therapy.

## Supplementary Materials

The following are available online at https://www.mdpi.com/2072-6694/12/6/1521/s1, Figure S1: Characterization and modeling parameters of COMSOL compression bioreactor model, Figure S2: Additional morphological analysis of ovarian cancer cells under compressive stimulus, Figure S3: Immunohistochemistry staining quantified for 72hr proliferation (ki67) and cell death response (casp-3) of OVCAR3 cells under compressive stimulus, Figure S4: G-Lisa analysis of CDC42 activation under compressive stimulus and in response to inhibitor treatment, Figure S5: Morphological response of ovarian cancer cells with chemotherapeutic drugs and CDC42 inhibitor treatment, Figure S6: Compression bioreactor system layout, Figure S7: Membrane characterization of the compression bioreactor. Table S1. RT-qPCR primer sequences.
