# Supplementary material for: Compressive Stimulation Enhances Ovarian Cancer Proliferation, Invasion, Chemoresistance, and Mechanotransduction via CDC42 in a 3D Bioreactor
Source: Cancers (Basel). 2020 Jun 10;12(6):1521. doi: 10.3390/cancers12061521 (PMC7352213; doi:10.3390/cancers12061521)
Supplement: Supplementary file 1 [file cancers-12-01521-s001.pdf]

Supplemental Materials:

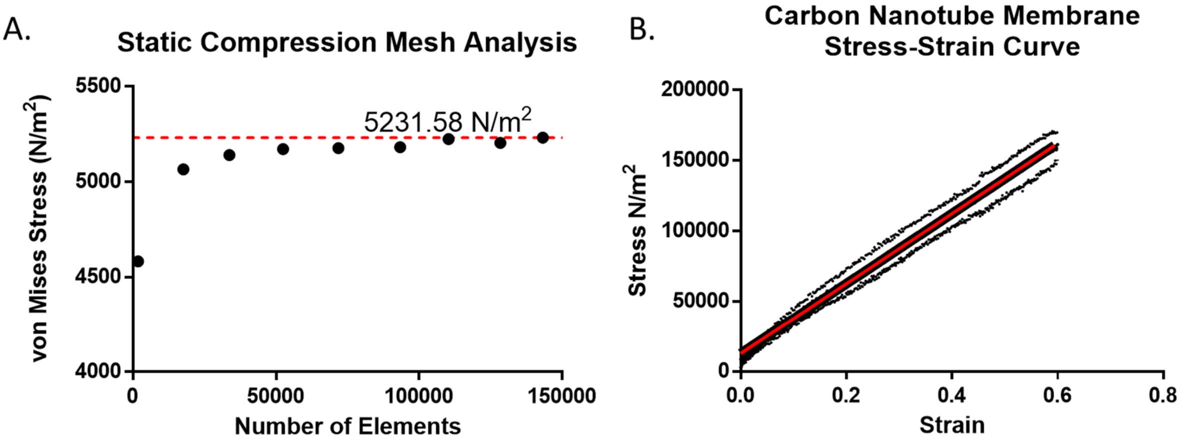

C.

Model component	Parameter	Value used
Hydrogel	Density	1030 kg/m³
	Youngs Modulus	26 kPa
	Shear Modulus	10 kPa
	Poisson's ratio	0.49
	Bulk Modulus	518 kPa
Membrane	Youngs Modulus	248 kPa
	Poisson's Ratio	0.49
	Density	960 kg/m³
	Bulk Modulus	41.4 GPa
	Shear Modulus	82.9 kPa

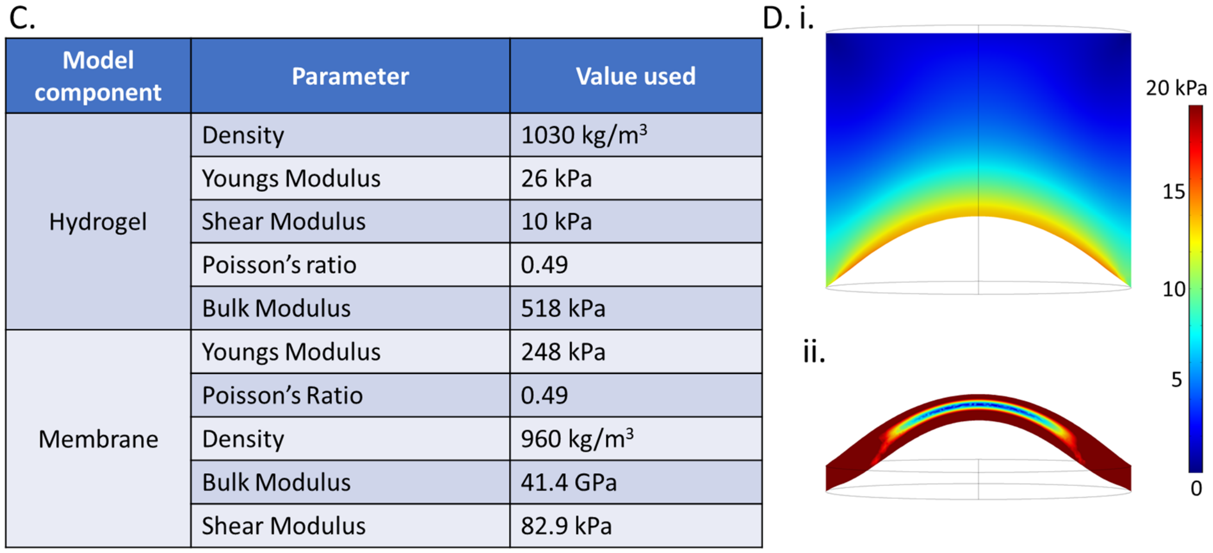

Supplemental Figure 1: Characterization and modeling parameters of COMSOL compression bioreactor model. **(A)** Mesh analysis of COMSOL model showing stress calculation is independent of the number of mesh elements. **(B)** Stress-strain curve with linear regression for the carbon-nanotube membrane. **(C)** Input parameter values used in compression bioreactor COMSOL model previously experimentally determined [10]. **(D)** Sample von Mises Stress output in the z-y plane of the hydrogel **(i)** and membrane **(ii)** under compressive stimulus.

COMSOL solid mechanics **Equation 1** was used for the computation of stress distribution within the deflecting membrane and hydrogel construct of the compression bioreactor.

$$-\nabla \cdot \sigma = Fv \quad (1)$$

Both the hydrogel and membrane were considered linear elastic and a boundary load was applied to the underside of the deflecting membrane at a pressure of 20 kPa.

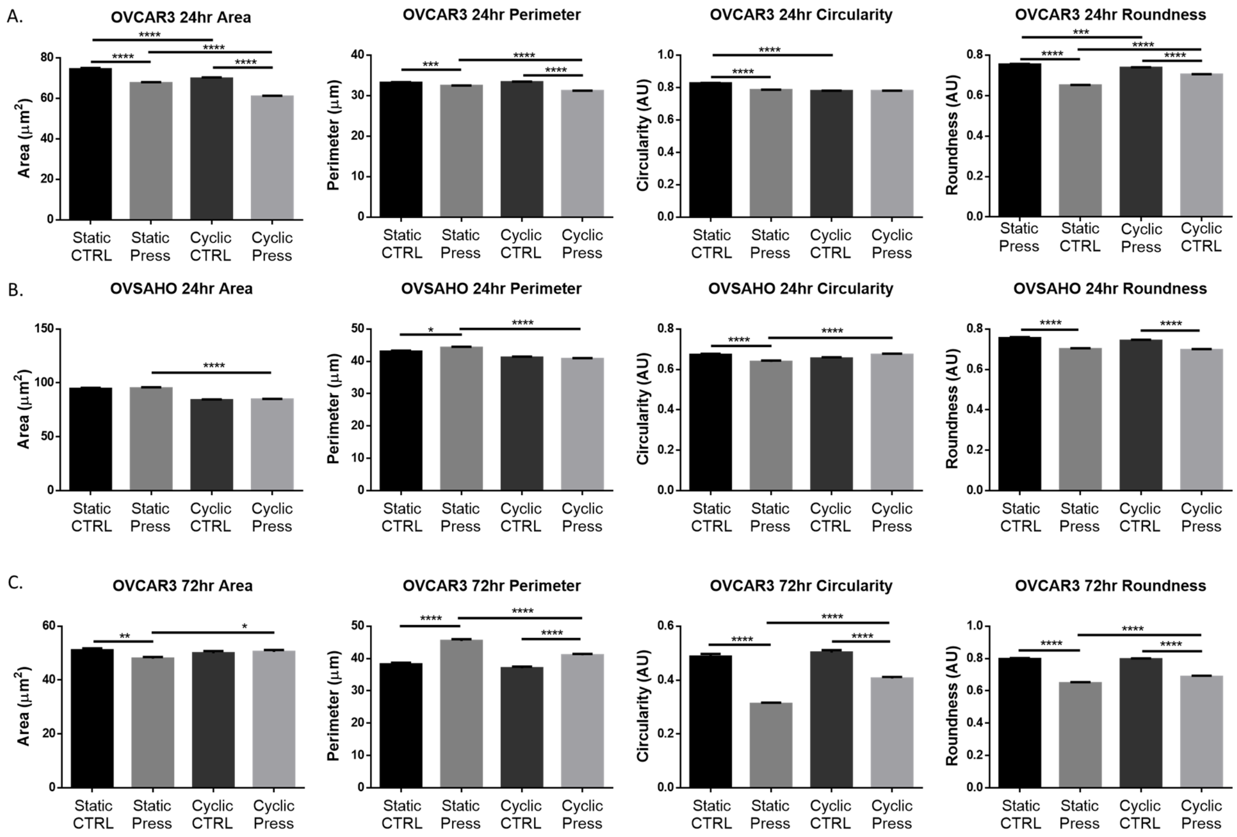

Supplemental Figure 2: Additional morphological analysis of ovarian cancer cells under compressive stimulus. Hematoxylin and eosin stains were quantified using ImageJ to encompass cell perimeters and calculate average area, perimeter, circularity, and roundness. Over 500 cells were quantified for each condition (One-way ANOVA, *p<0.1, **p<0.01, *** p<0.001, **** p<0.0001). **(A)** OVCAR3 cells stimulated for 24 hours of cyclic, static, or control conditions. **(B)** OVSAHO cells stimulated for 24 hours of cyclic, static, or control compression. **(C)** OVCAR3 cells stimulated for 72 hours.

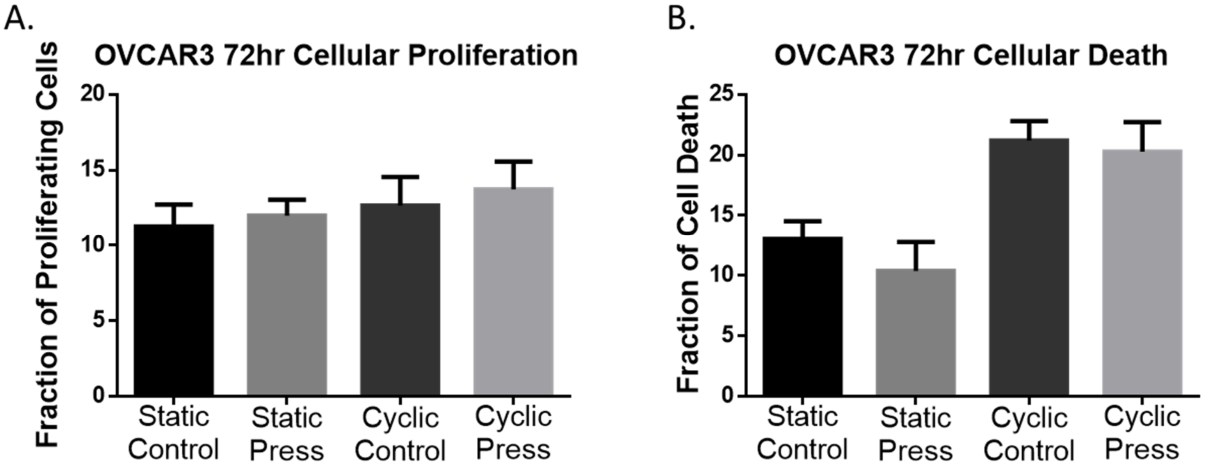

Supplemental Figure 3: Immunohistochemistry staining was quantified for **(A)** 72hr proliferation (ki67) and **(B)** cell death response (casp-3) of OVCAR3 cells under compressive stimulus. (t-test, *p<0.1, **p<0.01, *** p<0.001, **** p<0.0001).

Supplemental Table 1: RT-qPCR primer sequences

RT-qPCR Gene	Forward Primer	Reverse Primer
CDC42	5'- GTT CCC CAT CTG GTG CTC TTA G -3'	5'- CAC CAC CCC TCG TAT TTC CTC T -3'
OCT4	5'- GGG AGA TTG ATA ACT GGT GTG TT -3'	5'- GTG TAT ATC CCA GGG TGA TCC TC -3'
ABCB1	5'- GAG CCT ACT TGG TGG CAC AT -3'	5'- TCC TTC CAA TGT GTT CGG CA -3'
ABCG2	5'- TGA GCC TAC AAC TGG CTT AGA -3'	5'- CCC TGC TTA GAC ATC CTT TTC AG -3'

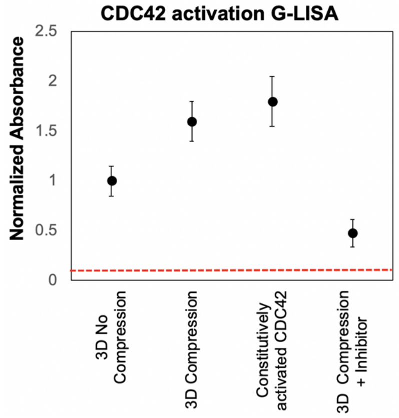

Supplemental Figure 4: G-Lisa analysis of CDC42 activation under compressive stimulus and in response to inhibitor treatment. Samples were normalized to the no compressive stimuli control. The constitutively active CDC42 provided in the G-lisa kit served as a positive control. The red dotted line indicates background absorbance. Maximum reduction of CDC42 activation (70%) was found with 100 µM ML141 treatment (denoted

as ‘3D compression+ Inhibitor’), and was used in compressive stimuli-inhibitor treatment experiments.

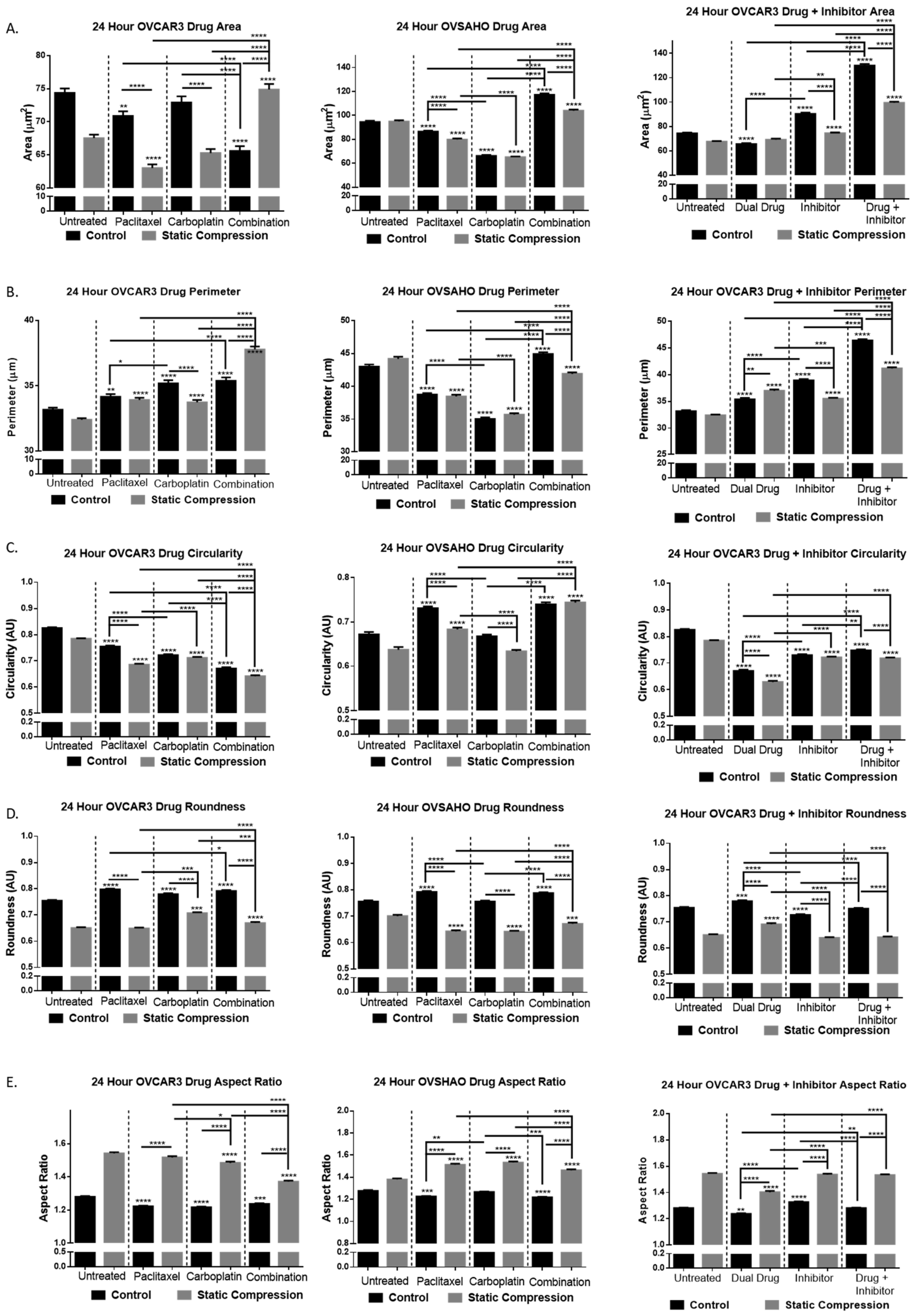

Supplemental Figure 5: Morphological response of ovarian cancer cells with chemotherapeutic drugs and CDC42 inhibitor treatment. Hematoxylin and eosin stains were quantified using ImageJ

to encompass cell perimeters and calculate average area, perimeter, circularity, roundness, and aspect ratio. Stars above bars indicate significance with respect to untreated control/compression conditions, respectively. Over 500 cells were quantified for each condition (One-way ANOVA *$p<0.1$, **$p<0.01$, *** $p<0.001$, **** $p<0.0001$).

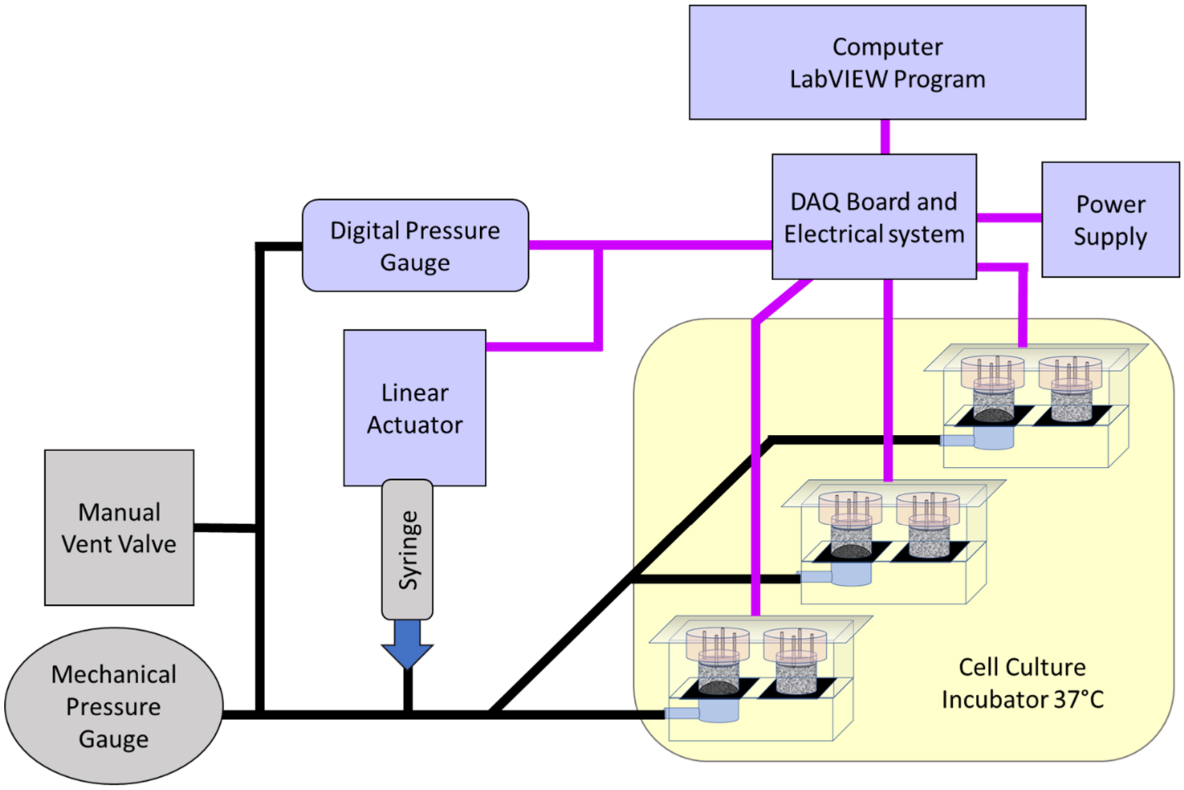

Supplemental Figure 6: Compression bioreactor system layout. Purple lines indicate electrical or signaling connections and black lines indicate air-pressure tubing connections. Air pressure changes were driven by the LabVIEW program which controlled linear actuator movement of the syringe. The three compression bioreactors were housed in the cell culture incubator and pressure changes were monitored through both mechanical and digital pressure gauges. Membrane deflection was monitored through a change in resistivity via the DAQ board system.

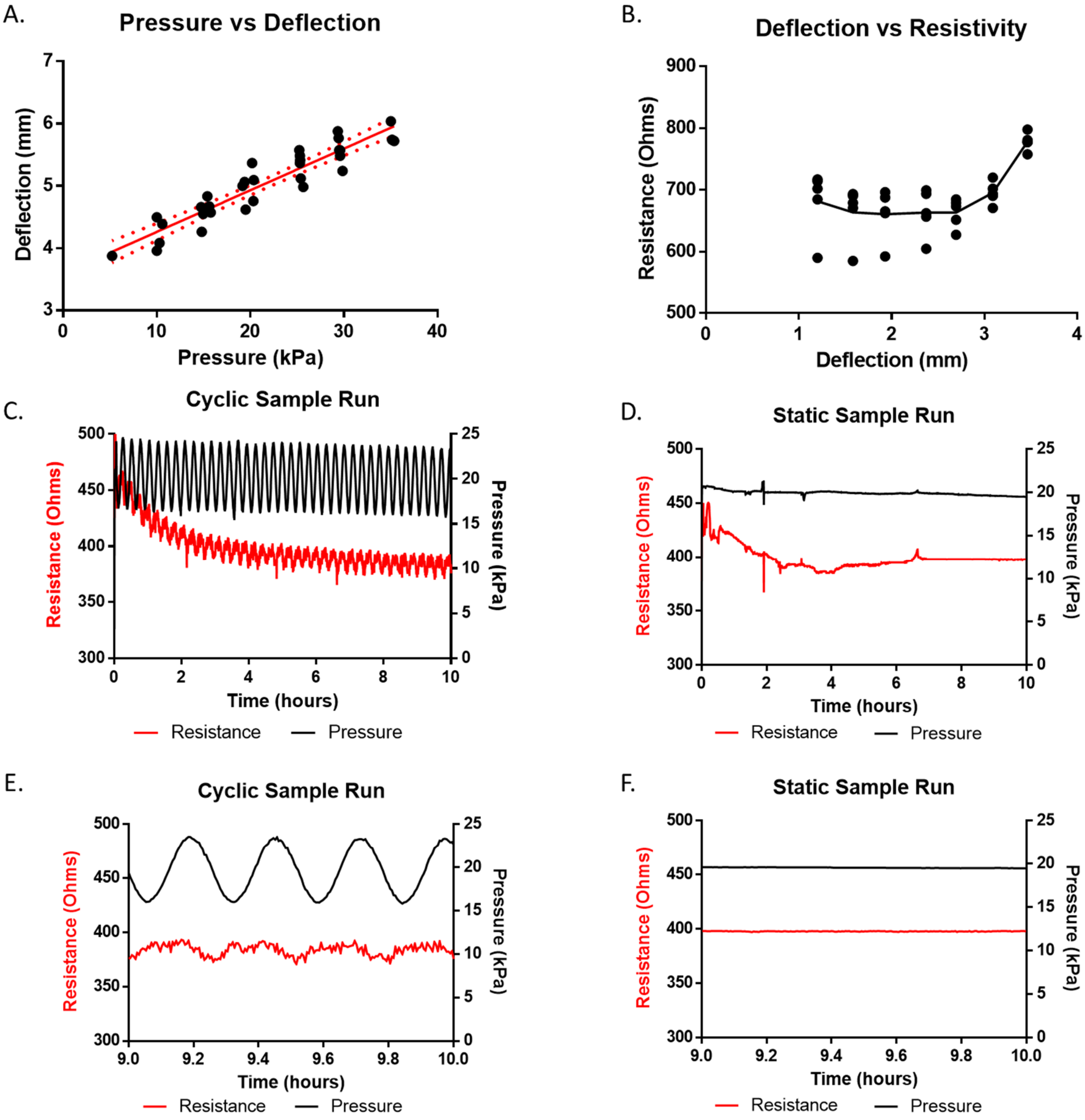

Supplemental Figure 7: Membrane characterization of the compression bioreactor. **(A)** Pressure vs deflection of the carbon nanotube membrane ranging from 5 to 35 kPa. **(B)** Deflection vs resistivity change of the carbon nanotube membrane. **(C-F)** change in resistivity over time for **(D and F)** sample static run and **(C and F)** a cyclic static run.
